# Distribution of microbes and antimicrobial susceptibility in patients with diabetic foot infections in South China

**DOI:** 10.3389/fendo.2023.1113622

**Published:** 2023-01-24

**Authors:** Wei Liu, Liying Song, Wei Sun, Weijin Fang, Chunjiang Wang

**Affiliations:** Department of Pharmacy, The Third Xiangya Hospital, Central South University, Changsha, Hunan, China

**Keywords:** diabetic foot infection, diabetic foot ulcer, microbes, antimicrobial susceptibility, diabetes

## Abstract

**Background:**

To investigate the distribution of microbes and drug susceptibility in patients with diabetic foot infections (DFI) and provide guidance for clinical empirical treatment and the rational selection of antibacterial drugs.

**Methods:**

Retrospective analysis of the pathogenic bacterium distribution and antimicrobial susceptibility isolated from 581 DFI patients with different Wagner grades.

**Results:**

The 534 positive samples included 473 cases (88.58%)) of monomicrobial infections and 61 cases (11.42%) of polymicrobial infections before antibiotic therapy. A total of 656 strains were cultivated, including 387 (58.99%) strains of gram-positive organisms (GPOs), 235 (35.82%) gram-negative bacilli (GNB), and 21 (3.20%) fungal strains. Polymicrobial infections mainly occurred in patients with Wagner grade 3-4 ulcers. GPOs were predominant in Wagner grades 1-3 (grade 1: 96.67%, grade 2: 76.52%, grade 3 62.81%), and the most common was Staphylococcus aureus (grade 1: 31.66%, grade 2: 33.04%, grade 3 35.53%). GNB were predominant in grades 4-5 (grade 4: 51.46%, grade 5:60%), and the most common GNB in Wagner grades 4-5 was *Proteus* (grade 4:27.88%, grade 5: 42.86%), while the most common GPO was *Enterococcus* (grade 4:34.48%, grade 5:25.00%). Staphylococcus (including MRSA) and Enterococcus were still highly sensitive to vancomycin, linezolid, and tigecycline. Most GNB were still highly sensitive to meropenem, tigecycline, ertapenem, and amikacin. *Proteus* was most sensitive to amikacin (97.14%), followed by meropenem (92%) and ertapenem (80%).

**Conclusion:**

The distribution of microbes and antimicrobial susceptibility in DFI patients varied with different Wagner grades. The most appropriate antimicrobial therapy should be selected based on the pathogen culture and antimicrobial susceptibility.

## Introduction

Diabetes is an important public health problem. The overall standardized prevalence of total diabetes using the American Diabetes Association (ADA) criteria was 12.8% in 2017 (1). There are estimated to be 129.8 million diabetes patients in mainland China (1). Diabetic foot is the leading cause of diabetes-related hospitalization, which is characterized by longer hospitalization, difficulty in treatment, and high medical costs (2, 3).

Diabetic foot infection (DFI) is one of the most important causes of the deterioration, amputation and death of patients with diabetes and is also a common cause of increased hospitalization and medical expenses (4, 5). Patients with foot ulcers have a high incidence of infections, and 40% to 70% of them have had infections when they seek medical treatment (6). Studies from different countries have shown that different degrees of infection in patients with DFI lead to different pathogenic microorganism distributions and drug sensitivities (7–9). Current studies have found that the microbial distribution of diabetic foot infections varies in different seasons in different countries (10–13). China is a vast territory, and types of diabetic foot bacterial infections are different in different regions (14, 15). Nevertheless, no multicenter studies have been performed to assess the microbial distribution of patients with DFI in China. In this study, we analyzed the clinical characteristics, pathogen distribution, and antimicrobial susceptibility of different Wagner grades in diabetic foot patients to provide a reference for the antimicrobial treatment of DFI.

## Methods

### Patients

A total of 581 diabetic foot patients hospitalized in the Endocrinology Department of the Third Xiangya Hospital of Central South University from January 1, 2018, to December 31, 2021, were selected as the research subjects, and diabetic foot secretions were collected for microbial culture and drug sensitivity tests. Patients receiving antibiotics 7 days before admission were included. The author should ensure that the work described has been carried out in accordance with the Code of Ethics of the World Medical Association (Declaration of Helsinki) for experiments involving humans.

### Specimen collection

Patients with DFI should be treated with sterile curette or scalpel before receiving antibiotic treatment, and then clean the wound with sterile saline. The process of wound sample collection was strictly aseptic. For patients with superficial ulcers, sterilized saline cotton swabs were dipped into the secretions or pus at the bottom of the ulcer for collection, and attention was paid to avoid contamination of the skin around the wound. For patients with deep ulcer and foot gangrene, the deep ulcer secretions or pus were collected by probe after debridement. The samples were immediately sent to the laboratory microbiology laboratory for aerobic bacteria, anaerobic bacteria, fungal culture and drug sensitivity tests.

### Microbiological assessment

The secretions were inoculated with blood, chocolate, and MacConkey agars plates for aerobic culture. The inoculated medium was incubated in a 5% CO_2_ incubator at 35°C for 24-48h. According to the morphology and gram staining characteristics of the colonies, the bacterial identification was carried out by the automatized VITEK 2 Compact system (bioMerieux, France). Kirby-Bauer, VITEK-2 Compact automatic system methods were used to test drug sensitivity. For anaerobic bacteria identification, samples were immediately inoculated on Columbia blood agars plates and Brucella agar plates, and were quickly incubated in an anaerobic incubator at 35°C for 48-72h. The distribution of specific strains was observed, and the strain types were determined with the aid of API20A kit. Drug sensitivity test was performed by using Kirby-Bauer method.

The selection of antimicrobial drugs for various types of bacterial susceptibility testing and the determination of the susceptibility results usually refer to the guidelines of the American Association for Clinical and Laboratory Standardization (CLSI) (16). MDR strains are defined according to the consensus issued by the European Centre for Disease Prevention and Control (ECDC) and the Centers for Disease Control and Prevention (CDC) in 2012 (17).

### Diabetic foot ulcer grade

The Wagner grade classification is as follows (18): 1) Grade 0: there are risk factors for foot ulceration, but no ulcer; 2) Grade 1: superficial ulcers of the feet without signs of infection; 3) Grade 2: Ulcer extension to the ligament, tendon, joint capsule, or deep fascia without abscess or osteomyelitis; 4) Grade 3: deep infection, bone lesions or abscess; 5) Grade 4:localized gangrene (toe, heel or foot); and 6) Grade 5: fully infected foot. The course of ulcer disease is based on acute and chronic wounds. Chronic ulcers refer to ulcers that have not improved after 4 weeks of treatment or have not been cured within 8 weeks (19).

### Statistics

All data were analyzed using SPSS 21.0 software (SPSS, Inc., Chicago, IL,USA). The measurement data are expressed as the mean ± standard deviation, and count data are expressed as a percentage [n (%)]. The chi-square test (χ2) was used for comparison between groups, and p <0.05 was considered statistically significant.

## Results

### Basic characteristics of patients

We included 704 secretion culture results from 581 DFI patients, including 65.06% (378/581) male patients and 34.94% (203/581) female patients. Among these patients, there were 573 (98.62%) patients with type 2 diabetes, 7 (1.20%) patients with type 1 diabetes, and 1 (0.17%) patient with latent autoimmune diabetes in adults (LADA).

The mean age of the patients was 61.29 ± 11.5 years, and 57.48% (334/581) of patients were aged 60 years and older. The mean duration of diabetes was 10.45 ± 6.84 years. Patients with different Wagner grades and wound conditions had different average hospital stays. The duration of hospitalization was similar between acute ulcers and chronic ulcers (χ^2 =^ 0.352, p=0.425). Of the 571 patients, 109 (19.09%) had good glycemic control (HbA1c ≤ 7%), 90 (15.76%) had normal glycemic control (HbA1c 7.1-8), and 372 (65.15%) had poor glycemic control (**Table 1**).

**Table 1:** Clinical characteristics of patients.

Parameters	Variable	Values (%)
Gender	Male Female	378 (65.06) 203 (34.94)
Age(years)	<40 40-50 50-60 60-70 70-80 >80	16 (2.75) 74 (12.74) 157 (27.02) 183 (31.50) 126 (21.69) 25 (4.30)
Type of diabetes	Type 1 LADA Type 2	7 (1.20) 1 (0.17) 573 (98.62)
Duration of diabetes (years)		10.45 ± 6.84
Duration of hospital stay (days)	Wagner 1 Wagner 2 Wagner 3 Wagner 4 Wagner 5 Duration of ulcer ≤ 4 weeks Duration of ulcer > 4 weeks Average length of hospital stay	11.84 ± 4.62 14.07 ± 6.41 21.43 ± 13.28 24.13 ± 15.94 23.68 ± 13.08 20.02 ± 14.02 20.38 ± 12.99 20.22 ± 13.38
Complication*	Peripheral neuropathy Nephropathy Peripheral vascular Retinopathy	530 (91.22) 265 (45.61) 353 (60.75) 319 (54.90)
HbA1c (%)	≤7 7.1∼8 8.1∼9 >9	109 (19.09) 90 (15.76) 89 (15.59) 283 (49.56)
Site of ulcers	Two feet Left foot Right foot	115 (19.79) 249 (42.86) 217 (37.35)

*Peripheral neuropathy, nephropathy, peripheral vascular and retinopathy were defined as chronic microvascular complications of diabetes. LADA, latent autoimmune diabetes in adult.

### Pathogen distribution

Before antimicrobial treatment, a total of 534 of the 581 secretion samples (91.57%) cultured pathogens (bacteria/fungi), of which 473 (88.58%) had monomicrobial infections and 61 (11.42%) had polymicrobial infections (number of microorganisms ≥ 2). A total of 20 positive bacteria and 12 negative bacteria were cultured after antibacterial treatment. A total of 656 microorganisms were cultured, including 387 (58.99%) gram-positive organisms (GPO), 235 (35.82%) gram-negative bacteria (GNB), and 21 fungal strains (3.20%). The detection rates of microorganisms were different in different Wager grades (χ^2^ = 9.531, p = 0.049), and multi-pathogen infections mainly occurred in patients with Wager grade 3-4 ulcers. GPO were predominant in Wagner grades 1-3 (grade 1: 96.67%, grade 2: 76.52%, grade 3 62.81%), and the most common was Staphylococcus aureus (grade 1: 31.66%, grade 2: 33.04%, level 3 35.53%). GNB were predominant in grades 4 - 5 (grade 4: 51.46%, grade 5:60%). The most common GNB in Wagner grades 1 -5 was Proteus (100% in grade 1, grade 2: 36.36%, grade 3: 32.43%, grade 4: 27.88%, grade 5: 42.86%), and the most common GPO was *Enterococcus* (grade 4: 34.48%, grade 5: 25.00%). The detection rate of fungi was 3.36%, mainly distributed in Wagner grade 2, including Candida albicans (33.33%), Candida tropicalis (33.33%), Candida parapsilosis (19.05%) (**Table 2**).

**Table 2:** The distribution of pathogenic bacteria was detected in DFI with different Wagner grades.

		Before antibiotic therapy (%)					After antibiotic therapy
Wagner	1	2	3	4	5	Total	
Total samples	31 (5.50)	122 (21.63)	213 (37.77)	169 (29.96)	29 (5.14)	564	109
Positive samples	28 (90.32)	102 (83.61)	199 (93.43)	160 (94.67)	28 (96.55)	517 (91.67)	32 (29.36)
Total strains	30	115	242	202	35	624	32
single pathogens	27 (87.10)	91 (74.59)	173 (81.22)	138 (81.66)	27 (93.10)	456 (80.86)	32 (100)
Multiple pathogens	1 (3.23)	11 (9.02)	26 (12.21)	22 (13.02)	1 (3.45)	61 (10.82)	0
MDR	9 (3.42)	38 (14.45)	97 (36.88)	100 (38.02)	19 (7.22)	263 (42.14)	8 (25.00)
Gram-positive bacteria	29 (96.67)	88 (76.52)	152 (62.81)	87 (43.07)	12 (34.29)	368 (58.97)	19 (59.38)
Staphylococcus aureus	11 (36.66)	38 (33.04)	54 (35.53)	29 (14.36)	3 (8.57)	137 (26.36)	2 (10.53)
Other Staphylococcus	12 (40.00)	29 (25.22)	42 (17.36)	19 (9.41)	4 (11.43)	106 (28.80)	7 (36.84)
MRSA	5 (16.67)	8 (6.96)	21 (8.68)	15 (7.43)	2 (5.71)	50 (13.59)	2 (10.53)
MRSE/MRSH	3 (10.00)	13 (11.30)	32 (13.22)	12 (5.94)	3(8.57)	61 (16.58)	5 (26.32)
Streptococcus	2 (22.22)	8 (9.09)	15 (9.87)	8 (9.20)	1 (8.33)	34 (9.24)	0
Enterococcus	2 (22.22)	10 (11.36)	34 (22.37)	30 (34.48)	3 (25.00)	79 (21.46)	8 (25.00)
Gram-negative bacteria	1 (3.33)	22 (19.13)	74 (30.58)	104 (51.46)	21 (60.00)	222 (35.57)	13 (40.62)
Klebsiella	0	6 (27.27)	14 (18.92)	14 (13.46)	2 (9.52)	36 (16.21)	0
Escherichia coli	0	2 (9.09)	9 (12.16)	14 (13.46)	4 (19.05)	29 (13.06)	3 (23.08)
Proteus	1 (100)	8 (36.36)	24 (32.43)	29 (27.88)	9 (42.86)	71 (31.98)	2 (15.38)
Enterobacter	0	3 (13.64)	7 (9.46)	14 (13.46)	3 (14.29)	27 (12.16)	0
Citrobacter	0	1 (4.55)	2 (2.70)	5 (4.81)	0	8 (3.60)	0
Morganella	0	1 (4.55)	4 (18.92)	5 (4.81)	1 (4.76)	11 (4.95)	2 (15.38)
Pseudomonas aeruginosa	0	0	4 (5.41)	10 (9.62)	0	14 (6.31)	2 (15.38)
Acinetobacter baumannii	0	0	2 (2.70)	1 (0.96)	0	4 (1.80)	3 (23.0)
Serratia marcescens	0	0	2 (2.70)	3 (2.88)	0	5 (2.24)	1 (7.69)
Bacteroides	0	0	3 (4.05)	3 (2.88)	0	6 (2.69)	0
Stenotrophomonas maltophilia	0	0	0	1 (0.96)	1 (4.76)	2 (0.90)	1 (7.69)
Myroides spp.	0	0	1 (1.35)	1 (0.96)	1 (4.76)	3 (1.35)	0
Aeromonas	0	1 (4.55)	0	3 (2.88)	0	4 (1.79)	0
ESBLs	0	4 (18.18)	8 (10.81)	11 (10.58)	3 (14.29)	26 (11.66)	1 (7.69)
Fungus	0	2 (1.74)	13 (5.37)	5 (2.48)	1 (2.86)	21 (3.20)	0

MDR, multidrug-resistant; ESBL, extended spectrum beta lactamase; MRSA, methicillin-resistant S. aureus; MRSH, methicillin-resistant Staphylococcus *haemolyticus*; MRSE, methicillin-resistant Staphylococcus *epidermidis*.

The culture positivity rates of MDR, extended-spectrum-lactamase (ESBL), and methicillin-resistant Staphylococcus aureus (MRSA) were 41.31% (271/656), 12.16% (27/222), and 37.41% (52/139), respectively. The highest detection rate of MDR at Wagner grade 4 was 38.02%. The highest detection rate of ESBL at Wagner grade 2 was 18.18%. Wagner grade 1 had the highest incidence rates of MRSA at 16.67%.

**Figure 1:**
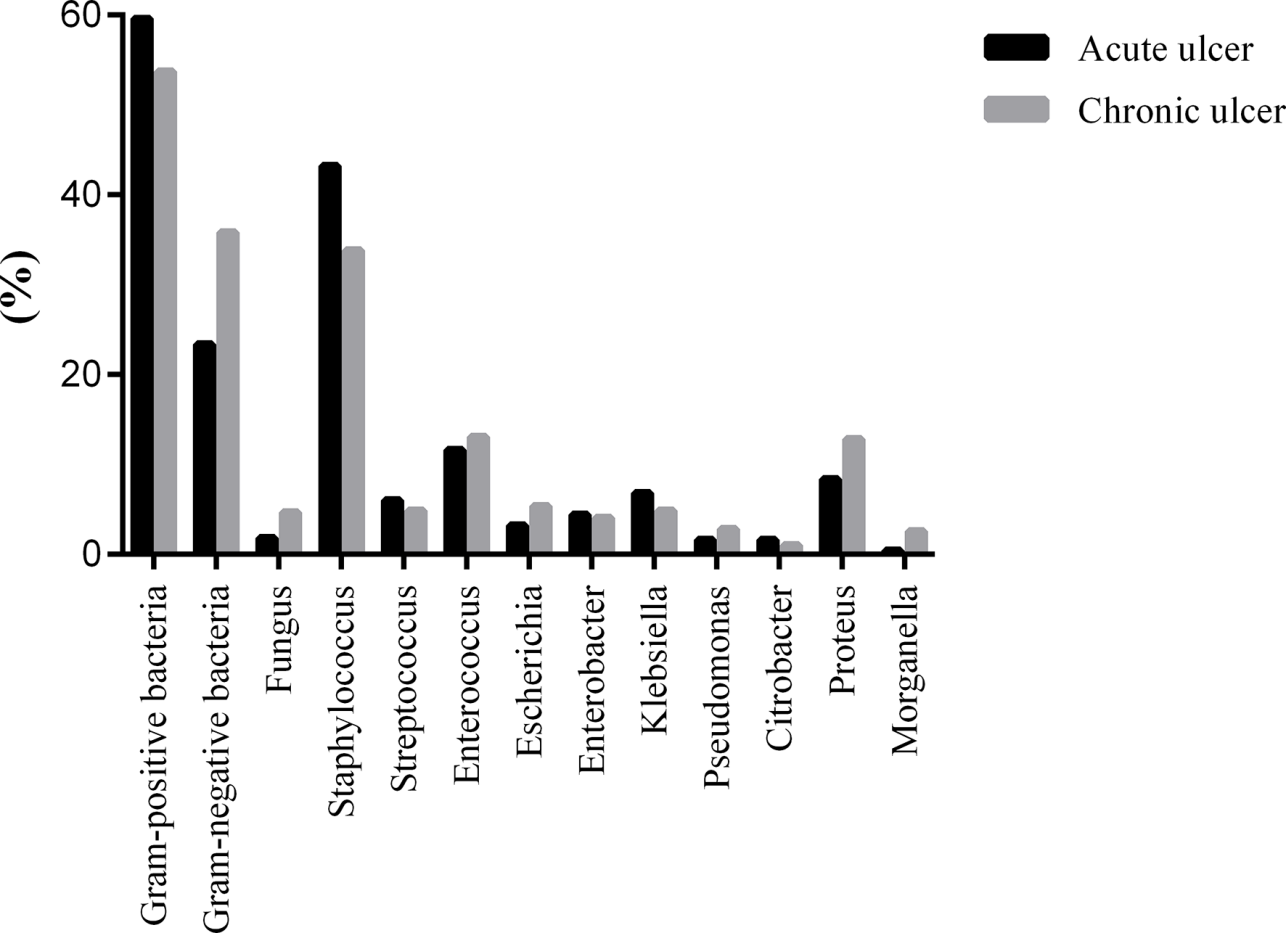
The distribution of pathogenic bacteria was detected in DFI with different duration of ulcer.

The positive rate of pathogenic bacteria in the acute ulcer stage of diabetic foot was 89.45%, of which the positive rate of GPO was 59.63% and that of GNB was 23.39%. The positive rate was 93.34% in the chronic ulcer stage, of which the positive rate of GPO was 53.72% and that of GNB was 35.81%; fungi accounted for 3.86%. The most common GPO in the acute and chronic ulcer period were Staphylococcus at 43.20% and 33.85%, followed by enterococci at 11.06% and 13.08%. Proteus was the most common GNB in the acute (8.40%) and chronic ulcer stages (12.82%) (**Figure 1**).

### Drug sensitivity testing

Staphylococcus was still highly sensitive to vancomycin, linezolid, and tigecycline. Among the 137 strains of Staphylococcus aureus, 2 strains were resistant to vancomycin and 1 strain was intermediary. Two strains were resistant to vancomycin and 3 strains were intermediary in the 37 strains of hemolytic Staphylococcus. Among 33 strains of Staphylococcus epidermidis, 1 strain was intermediary to vancomycin, and 1 strain was intermediary to tigecycline (**Table 3**). Staphylococcus aureus maintains a high sensitivity to linezolid (100.00%) and tigecycline (100.00%), followed by vancomycin (97.81%), rifampicin (96.35%), sulfamethoxazole (91.24%), and moxifloxacin (83.94%). Other antibacterial drugs were more than 50% sensitive, including clindamycin (51.09%), tetracycline (66.96%), erythromycin (51.09%), ciprofloxacin (71.43%), and levofloxacin (70.80%).

**Table 3:** Drug sensitivity results of gram-positive bacteria from diabetic foot patients.

	Staphylococcus aureus	Staphylococcus haemolyticus	Staphylococcus epidermidis	Enterococcus faecalis	Enterococcus faecium	Streptococcus
Total strains	137	37	33	68	9	34
Oxacillin (%)	62.77	5.56	9.09	—	—	–
Ampicillin (%)	—	0.00	—	95.59	11.11	90.00
Penicillin G (%)	4.38	100.00	0.00	95.38	11.11	90.00
Macrodantin (%)	94.83	90.63	90.00	94.44	50.00	80.00
Moxifloxacin (%)	83.94	29.73	51.52	84.31	14.29	66.67
Levofloxacin(%)	70.80	13.51	27.27	79.10	33.33	70.59
Ciprofloxacin (%)	71.43	5.88	0.00	76.79	12.50	60.00
Sulfamethoxazole(%)	91.24	45.95	39.39	—	—	–
Tetracycline(%)	66.96	43.75	50.00	16.36	14.29	33.33
Erythromycin (%)	51.09	5.56	18.18	1.47	0	20.00
Clindamycin(%)	51.09	30.56	33.33	—	0	28.00
Gentamicin (%)	91.97	30.56	72.73	97.14	28.57	–
Rifampicin (%)	96.35	75.00	90.91	—	—	–
Tigecycline (%)	100.00	100.00	96.67	100.00	100.00	100.00
Vancomycin(%)	97.81	86.49	96.97	95.52	100.00	100.00
Linezolid (%)	100.00	100.00	100.00	98.08	100.00	100.00
Quinupristin/dalfotristin (%)	99.21	93.94	9.09	100.00	71.43	83.33

**Figure 2:**
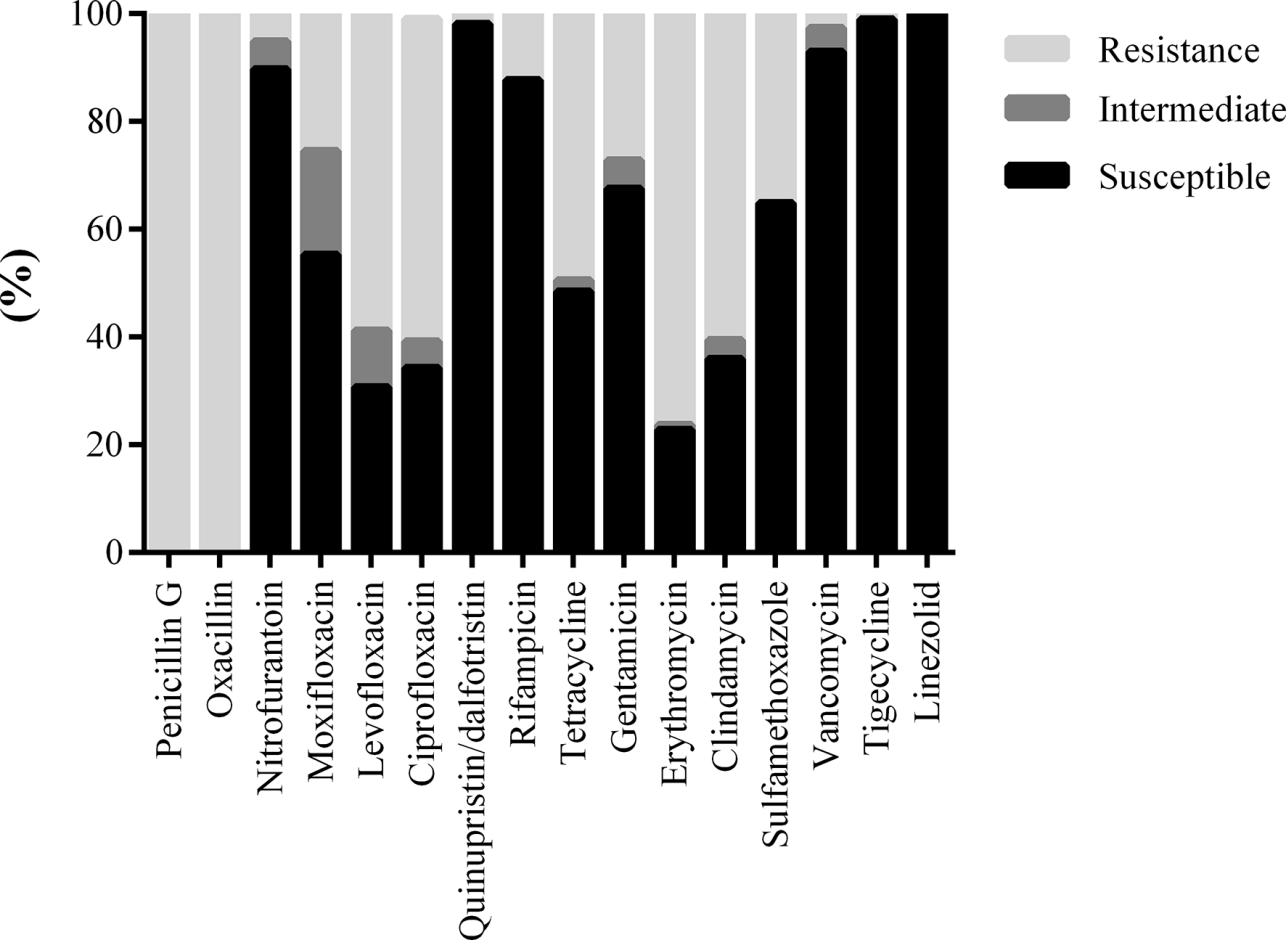
Drug sensitivity results of MRS from diabetic foot patients.

A total of 111 methicillin-resistant Staphylococcus (MRS) strains were cultured, of which 51 strains were MRSA. MRS maintains had the highest sensitivity to linezolid (100.00%), followed by tigecycline (98.99%), vancomycin (92.98%), and rifampicin (87.72%), and had high resistance to clindamycin (35.96%), levofloxacin (30.70%), ciprofloxacin (34.31%), and erythromycin (22.21%) (**Figure 2**). A total of 79 cases of Enterococcus were detected, of which Enterococcus faecium maintained 100% sensitivity to linezolid, tigecycline, and vancomycin and had high resistance to ampicillin (11.11%), penicillin G (11.11%), high-concentration gentamicin (28.57%), ciprofloxacin (12.50%), and levofloxacin (33.33%). Enterococcus faecalis was highly sensitive to tigecycline (100.00%), followed by linezolid (98.08%), vancomycin (95.52%), ampicillin (95.59%), penicillin G (95.38%), and high-concentration gentamicin (97.14%), and maintained more than 75% sensitivity to quinolones. Among these, two strains of Enterococcus faecalis were resistant to vancomycin and two were intermediary, and one was resistant to linezolid (**Table 3**).

**Figure 3:**
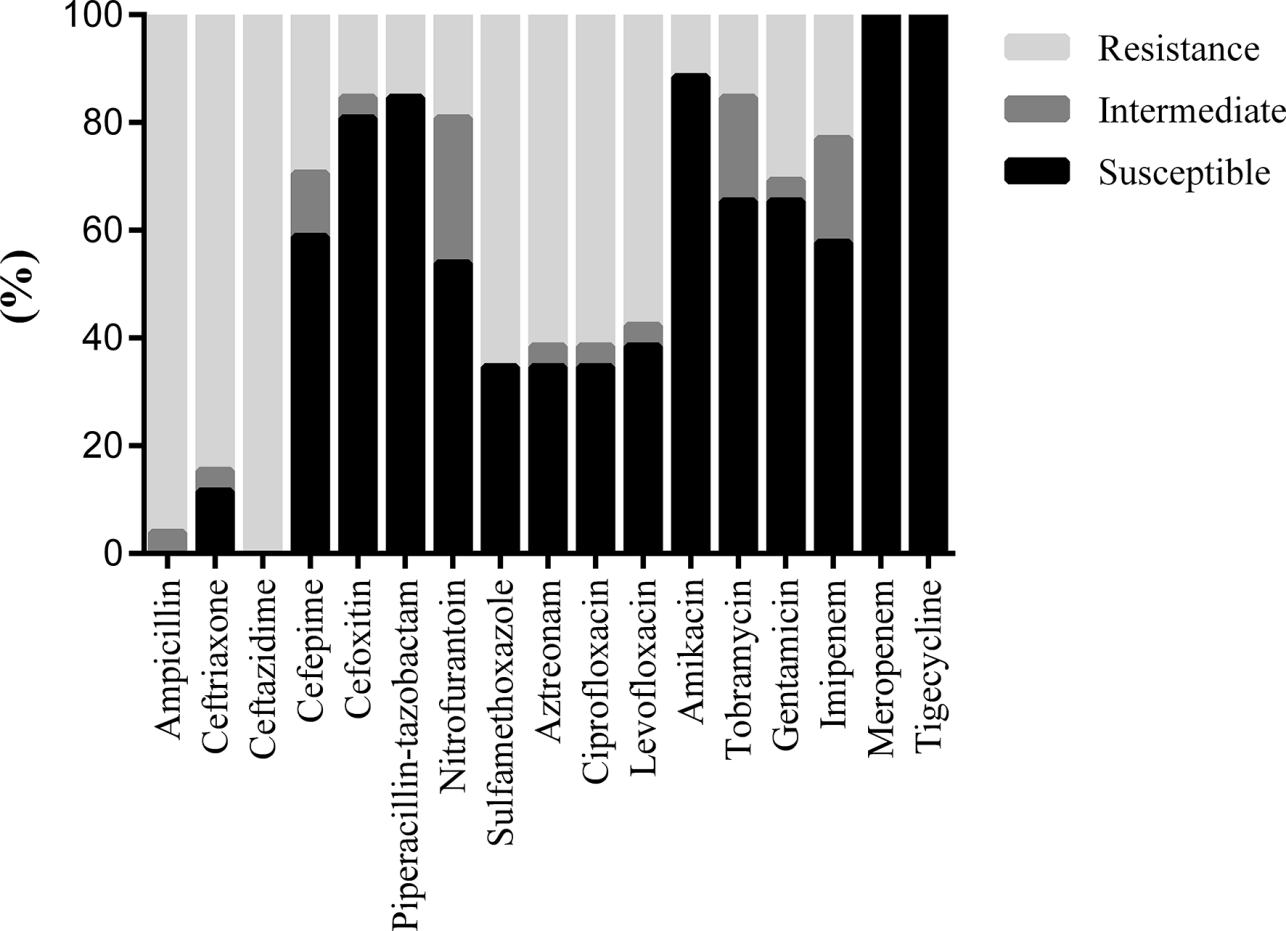
Drug sensitivity results of product ESBL bacteria from diabetic foot patient.

**Figure 4:**
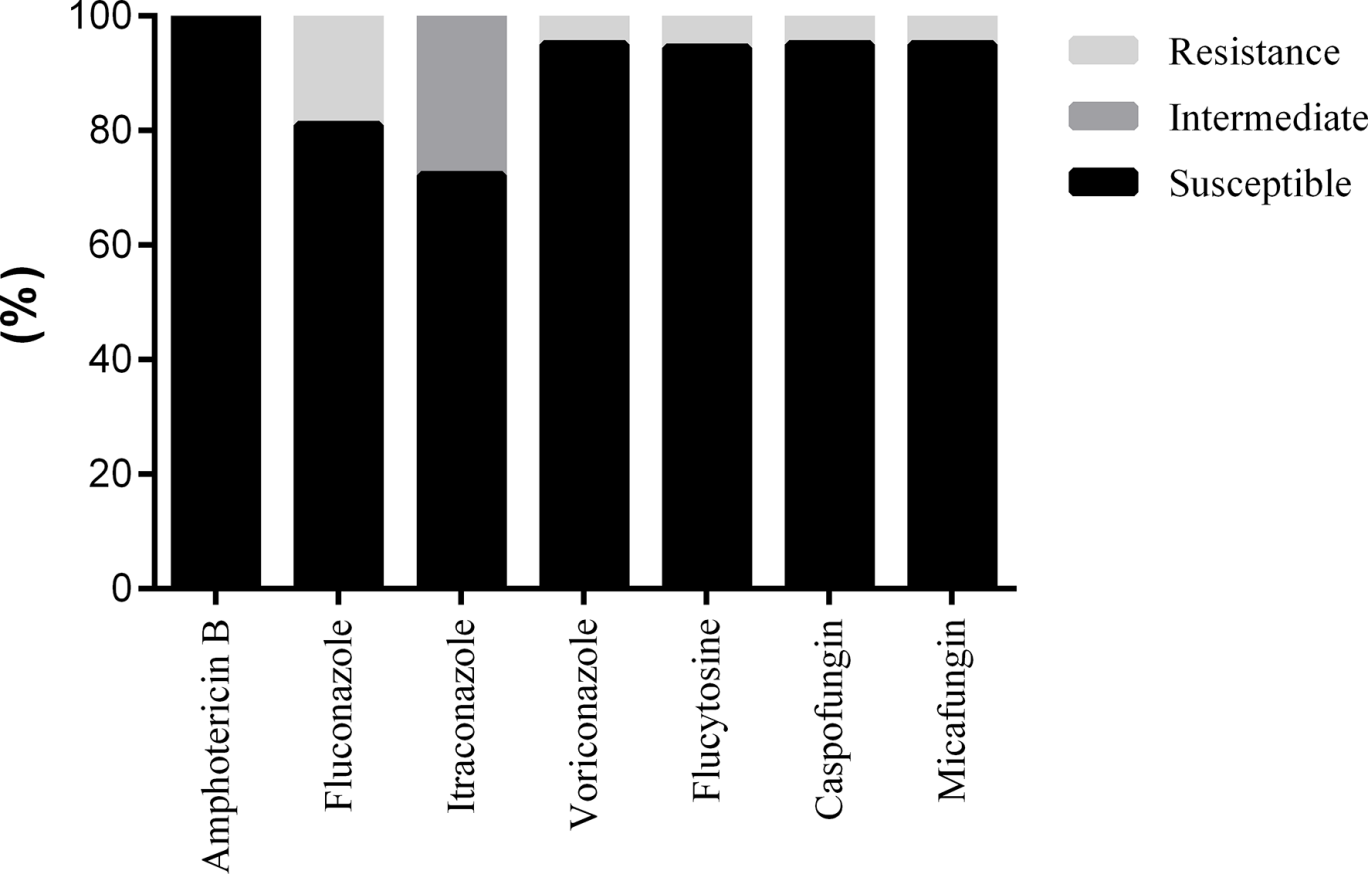
Drug sensitivity results of Fungus from diabetic foot patients.

Most GNB still maintained high sensitivity to meropenem, tigecycline, ertapenem, and amikacin (**Table 4**). The Proteus genus had the highest detection rate among GNB and the highest sensitivity to amikacin (97.14%), followed by meropenem (92%), ertapenem (80%), ceftazidime (77.27%), piperacillin and tazobactam (73.91%), levofloxacin (72.46%), aztreonam (72.46%), cefepime (71.43%), and gentamicin (71.21%) and was still highly resistant to tigecycline. A total of 26 ESBL-producing bacteria were detected, which remained 100% sensitive to tigecycline and meropenem, followed by amikacin (88.46%), piperacillin and tazobactam (84.62%), cefoxitin (80.77%), while the sensitivity to quinol was below 40% (**Figure 3**). The sensitivities of Candida to amphotericin B, caspofungin, micafungin, voriconazole, flucytosine, fluconazole, and itraconazole were 100.00%, 95.00%, 95.00%, 95.00%, 94.44%, 80.95%, and 72.22%, respectively (**Figure 4**).

**Table 4:** The drug sensitivity results of gram-negative bacteria from diabetic foot patients.

	Escherichia coli	Serratia	Klebsiella	Enterobacter	Proteus	Pseudomonas	Acinetobacter baumannii	Morganella	Citrobacter
Total strains	32	6	36	27	73	17	6	13	8
Macrodantin (%)	64.29	25.00	35.71	12.50	3.17	0.00	0	0	–
Ampicillin(%)	9.38	0	0.00	0.00	15.63	0.00	0	0	100.00
Ampicillin-sulbactam (%)	22.22	0	50.00	100.00	53.85	11.11	0	0	–
Piperacillin-tazobactam (%)	75.00	25.00	91.67	44.00	73.91	53.33	20.00	58.33	83.33
Sulfamethoxazole(%)	34.38	100.00	58.82	54.17	47.06	0.00	50.00	41.67	0
Cefazolin (%)	66.67	0	100.00	0.00	25.00	0.00	–	0	100.00
Cefoxitin (%)	64.29	0	86.67	0.00	66.15	0.00	0	33.33	–
Ceftriaxone (%)	35.71	33.33	73.33	47.37	62.50	8.33	0	58.33	71.43
Ceftazidime (%)	57.14	74.00	86.96	45.45	77.27	100.00	0	50.00	12.50
Cefepime (%)	55.00	80.00	83.33	82.35	71.43	80.00	0	60.00	0
Levofloxacin(%)	40.63	50.00	75.00	51.85	72.46	81.25	33.33	83.33	0
Ciprofloxacin (%)	40.63	50.00	69.44	56.00	63.77	81.25	20.00	50.00	0
Aztreonam (%)	48.39	50.00	69.44	40.74	72.46	–	0	63.64	–
Amikacin (%)	90.63	100.00	91.43	96.00	97.14	100.00	100.00	100.00	71.43
Tobramycin (%)	64.52	80.00	73.53	55.00	69.23	80.00	40.00	50.00	0
Gentamicin (%)	62.96	100.00	74.19	75.00	71.21	76.92	33.33	63.64	0
Meropenem(%)	95.00	100.00	100.00	95.24	92.00	100.00	0	100.00	–
Imipenem (%)	68.75	25.00	63.89	44.00	0.00	62.50	16.67	50.00	100.00
Ertapenem (%)	80.00	100.00	100.00	88.89	80.00	–	–	100.00	71.43
Tigecycline (%)	100.00	100.00	100.00	95.65	5.80	6.67	50.00	0	–

## Discussion

Diabetic foot can seriously affect patients’ daily life and work, reduce patients’ health and quality of life, and even threaten patients’ life safety. Diabetes increases the risk of cardiovascular disease, and diabetic foot ulcers (DFU) may further increase this risk. A meta-analysis found that DFU were associated with an increased risk of fatal myocardial infarction and fatal stroke (20). DFI is an important factor in the deterioration of diabetic foot (21). Treatment with antibiotics in DFI is imperative to improve outcomes. The initial treatment of diabetic infections is usually empirical and depends on the severity and extent of infection and any available microbiological data. With increase in age, S. aureus, Streptococci and Pseudomonas aeruginosa became more frequent. The proportion of mixed bacterial infection cases in elderly DFI patients was relatively high, and the drug resistance was higher than that in non-mixed infection patients (22). This may be related to the fact that elderly patients with DFI have underlying diseases, organ function decline, peripheral tissue oxygen supply and weak regeneration ability. In addition, some patients had received systemic or local antibiotics before admission, which further affected the distribution of pathogenic bacteria on the wound surface. Consistent with most studies (14, 23), DFI occurred in elderly male patients with type 2 diabetes, accompanied by some complications and poor blood glucose control.

The investigation found that GPO (59.75%) predominated in DFI, which was consistent with the survey results of DFI in southern China from 2009 to 2014, with GPO accounting for 54% (23), and different from studies in Southwest China, Beijing area and South India, where GNB accounted for 51%, 57.5%, and 51.4%, respectively (14, 24, 25). The most common GPO was Staphylococcus aureus, consistent with other reports (23, 24, 26–28). Proteus among the GNB was the most frequently isolated in our study, which was different from other reports, such as Pseudomonas aeruginosa (28–30) and Escherichia coli (15, 26). This study further demonstrated that DFI bacteria were different in different regions. Monomicrobial infections were the main cause, consistent with other studies from China (14, 24), accounting for 56.8% andw79.8%, and different from Pakistan and Kuwait, where polymicrobial infections accounted for 56.9% and 75%, respectively (26, 27).

In our study, The pathogen spectrum of DFI patients with high Wagner grade is mainly gram-negative bacilli and multimicrobial infection, especially in patients with Wager 3-4 ulcers, as previously reported (14, 15). Therefore, when using antibiotics empirically, DFI patients with Wagner ≥3 should use a combination of antibiotics or broad-spectrum antibiotics to ensure the simultaneous coverage of GPO and GNB.

In addition, the GPO mainly changed from Staphylococcus aureus to Enterococcus with the increased Wagner grade. Enterococci often appeared in patients with low immunity and could participate in the formation of biofilms (29, 31). The positive rates of pathogenic bacteria in the acute ulcer stage and the chronic ulcer stage of the diabetic foot were similar, and both were mainly GPO. After antimicrobial treatment, the positive rate of bacteria was significantly reduced, and GPO were still predominant, which was different from Southwest China (14).

Increased resistance of pathogenic bacteria is an important problem in the treatment of DFI. The study found that MDR organisms were very common in patients with DFI, which was in accordance with earlier studies (30, 32). In our research, Staphylococcus is the most common MDR organisms, similar to previous studies, followed by Proteus mirabilis (15, 24). The emergence of MDR organisms increases the risk of amputation, mortality, additional morbidity, hospital stay duration and costs of management in patients with DFI (30, 33–35). These instructions indicated that we should adjust antimicrobial drugs in a timely manner based on drug sensitivity and therapeutic effects.

In the present study, the culture positivity of MRSA infection was 37.41%, significantly higher than in previous studies from China (23, 27). Previous studies reported that MRSA is the main cause of suppurative skin and soft tissue infections (36). MRSA infections prolong wound healing times and hospitalization stays, increase the need for surgical procedures, and result in treatment failure (35). Long-term (more than 6 months) antibiotics, long foot wound duration, previous hospitalization history, high blood pressure, anemia, chronic osteomyelitis, and history of MRSA infection have been recognized as the predictive risk factors (37). Our region has a high infection rate of MRSA. According to IDSA guidelines, it has been necessary to cover MRSA regularly in patients with a previous history infection of MRSA, high local prevalence of MRSA, and very severe infection (38). Our research found that GPO, including staphylococcus, enterococcus, and streptococcus, were highly sensitive to vancomycin, linezolid, and tigecycline and were resistant to erythromycin and clindamycin. Staphylococcus aureus and Enterococcus faecalis were still more than 70% sensitive to fluoroquinolone, as previously reported from China (23, 25). Additionally, Bravo-Molina et al. found that fluoroquinolone antibiotics were the most sensitive antibiotics for GPO (19). MRS was highly sensitive to linezolid, tigecycline, vancomycin, rifampicin. For Enterococci, Enterococcus faecium had lower positive rates and higher resistance to ampicillin, penicillin G, high-concentration gentamicin, and fluoroquinolones compared with Enterococcus faecalis, which was consistent with the 2018 CHINET bacterial resistance monitoring results (33).

This study showed that GNB remained highly sensitive to meropenem, tigecycline, and amikacin, but previous studies showed a different pattern of susceptibility (14). Compared to other studies, we found that the prevalence of ESBL-producing isolates was higher in patients with DFIs (14). ESBL-producing Enterobacteriaceae showed higher susceptibility to meropenem, tigecycline, piperacillin-tazobactam, and amikacin and a high resistance rate to fluoroquinols. The ESBL-producing bacteria had fewer effective antibacterial drugs and could increase the length of hospital stay in patients with DFI (39).

## Conclusion

In conclusion, this study provides a reference for the local bacterial distribution, antimicrobial sensitivity and empirical treatment. In the treatment of DFI, microbiology examination should be performed in a timely manner, and effective antibiotics should be selected to improve the clinical outcomes of DFI patients according to the severity of ulcers and infections, the risk factors of drug-resistant bacteria and antimicrobial susceptibility.

## Data availability statement

The original contributions presented in the study are included in the article/supplementary material. Further inquiries can be directed to the corresponding authors.

## Ethics statement

Ethical review and approval was not required for the study on human participants in accordance with the local legislation and institutional requirements. Written informed consent for participation was not required for this study in accordance with the national legislation and the institutional requirements.

## Author contributions

WL and CW managed the study database, conducted the analysis, and wrote the first draft of the manuscript. LS, WS, WF, and CW edited the manuscript. WL, LS, WS, WF, and CW reviewed the last version of the manuscript. All authors contributed to the article and approved the submitted version.
